# The first transcriptome sequencing and data analysis of the Javan mahseer (*Tor tambra)*

**DOI:** 10.1016/j.dib.2021.107481

**Published:** 2021-10-14

**Authors:** Melinda Mei Lin Lau, Leonard Whye Kit Lim, Hung Hui Chung, Han Ming Gan

**Affiliations:** a Faculty of Resource Science and Technology, Universiti Malaysia Sarawak, Kota Samarahan, Sarawak 94300, Malaysia; b GeneSEQ Sdn Bhd, Bandar Bukit Beruntung, Rawang, Selangor 48300, Malaysia; c Center for Integrative Ecology, School of Life and Environmental Sciences, Deakin University, Geelong, Victoria, Australia

**Keywords:** Transcriptome, Unigenes, Gene annotation, *Tor tambra*

## Abstract

The Javan mahseer (*Tor tambra*) is one of the most valuable freshwater fish found in *Tor* species. To date, other than mitogenomic data (BioProject: PRJNA422829), genomic and transcriptomic resources for this species are still lacking which is crucial to understand the molecular mechanisms associated with important traits such as growth, immune response, reproduction and sex determination. For the first time, we sequenced the transcriptome from a whole juvenile fish using Illumina NovaSEQ6000 generating raw paired-end reads. *De novo* transcriptome assembly generated a draft transcriptome (BUSCO5 completeness of 91.2% [Actinopterygii_odb10 database]) consisting of 259,403 putative transcripts with a total and N50 length of 333,881,215 bp and 2283 bp, respectively. A total count of 77,503 non-redundant protein coding sequences were predicted from the transcripts and used for functional annotation. We mapped the predicted proteins to 304 known KEGG pathways with signal transduction cluster having the highest representation followed by immune system and endocrine system. In addition, transcripts exhibiting significant similarity to previously published growth-and immune-related genes were identified which will facilitate future molecular breeding of *Tor tambra*.

**Specifications Table**

**Table:** 

Subject	Biological Sciences
Specific subject area	Omics: Transcriptomics
Type of data	Sequencing raw reads, assembly, Table, Figure, Graph
How data were acquired	Sequencing
Data format	Raw Reads (fastq), Assembly (fasta)
Parameters for data collection	Total RNA extracted from a whole specimen of fish fry was used for library preparation and sequencing.
Description of data collection	Total RNA extraction was performed using Wizol TriZol-like reagent (WizBio). The purified total RNA was subjected to mRNA enrichment using poly-T magnetic bead (NEB). The enriched mRNA was subsequently processed using NEB Ultra II RNA library preparation kit and sequenced on an Illumina NovaSeq6000 (2 × 150 bp)
Data source location	The sample fish fry in this study was provided by a fish breeder who claimed that it originated from the Pahang, Malaysia. We subsequently extracted the mitochondrial genes from the transcriptome and showed that this specimen indeed formed a monophyletic cluster with *Tor* spp described from Pahang, Malaysia (Fig. 1) [1].
Data accessibility	Raw data and final assembled contigs were deposited in the NCBI database under the Bioproject PRJNA727425 (https://www.ncbi.nlm.nih.gov/bioproject/PRJNA727425). Additional files such as BUSCO analysis output, GO annotation, KEGG annotation and COG annotation are available in the Zenodo database https://doi.org/10.5281/zenodo.4766490.

**Value of the Data**

• Transcriptome dataset from the Javan mahseer is useful to gain insight into transcription regulation and biomarker discovery for the subsequent improvement of this species for aquaculture purposes.
• High completeness of transcriptome dataset will aid in future phylotranscriptomic studies especially for fish taxonomist.
• The dataset is useful in facilitating genetic management for the conservation of remaining populations of mahseer in Malaysian rivers.

## 1 Data Description

Standard RNA sequencing was performed to generate the transcriptome assembly from Javan mahseer (*Tor tambra*). Sequencing and assembly results are summarized in Table 1. Coding region was extracted using TransDecoder generating 77,503 predicted non-redundant proteins [2]. The proteins were annotated using eggNOG mapper [3] that will perform mapping to the KEGG, GO and COG databases. The sequence length of each unigene ranged from < 300 bp to > 5000 bp (Fig. 2). The number of unigenes had shown a decreasing trend when the length increases. A total of 40,150, 42,644 and 61,616 unigenes were annotated to GO, KEGG and COG databases, respectively. A Venn diagram had illustrated the differences and commonalities of unigenes toward the three databases (Fig. 3). Among a total of 63,191 unigenes, COG databases had the highest number of matches (61,616 unigenes) while another 42,644 and 40,150 unigenes matched to KEGG and GO databases, respectively (Table 2). Overall, 32,317 (51.14%) unigenes were found to exhibit a significant match to all the three major databases with 50,405 unigenes (79.77%) portrayed significant match to at least one hit to these databases (Table 2).

**Table 1:** Transcriptome sequencing and assembly statistics.

Raw sequence reads	108,657,770 (16.29 Gb)
Number of contigs Total assembled contig length Contig N50 length Number of predicted proteins Total predicted protein length	278, 297 276, 327, 107 bp 1,922 bp 77,503 24,833,897 aa
BUSCO Completeness (Actinopterygii odb10)	
Actinopterygii odb10: Complete BUSCOs	84% (3055)
Complete and single-copy BUSCOs	18.7% (679)
Complete and duplicated BUSCOs	6.9% (250)
Missing BUSCOs	9.1% (335)

**Fig. 1:**
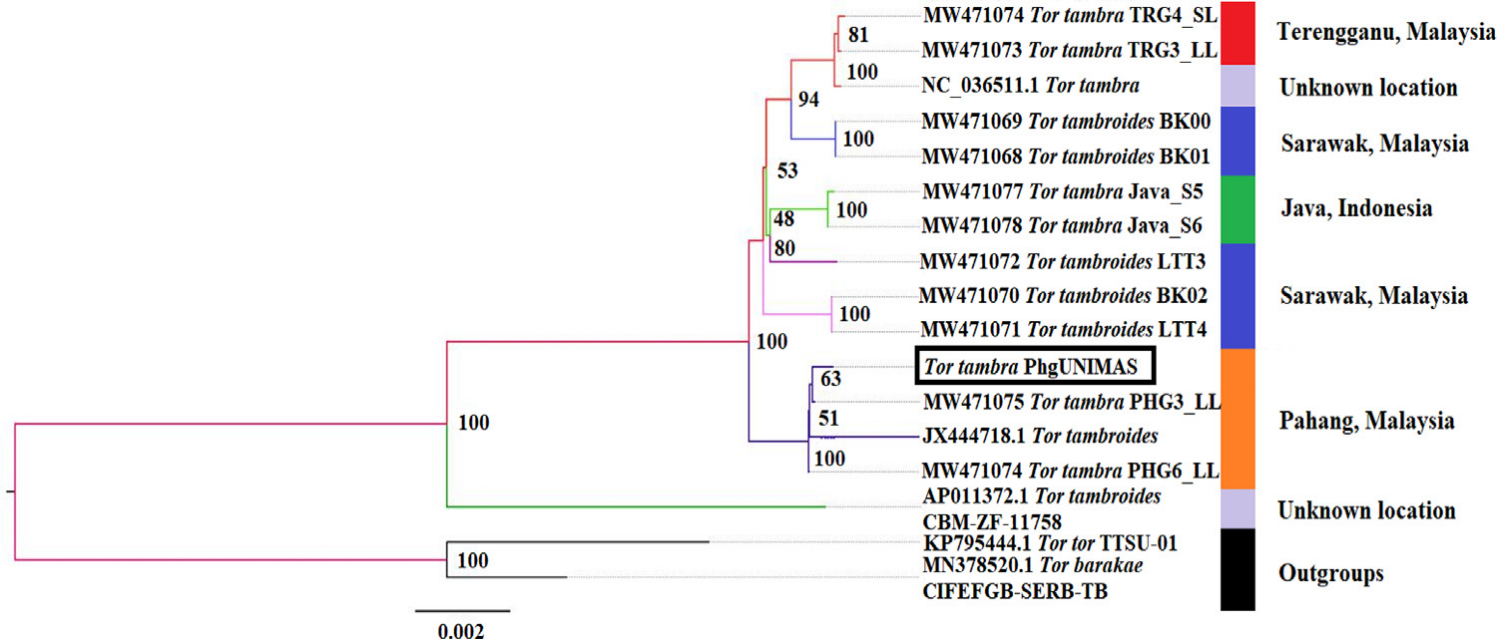
The maximum-likelihood phylogenetic tree constructed based on standard cytochrome oxidase I gene fragment with 1000 bootstrap replications, with the black bracket highlighted showing the sample fish fry involved in this study [1].

**Fig. 2:**
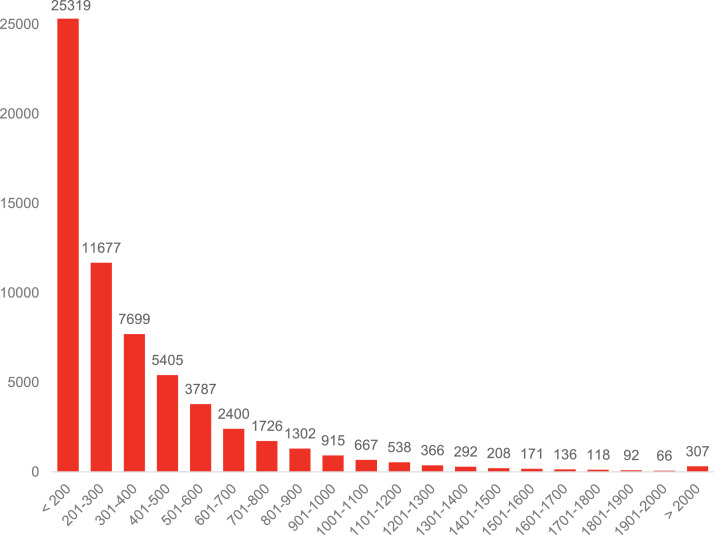
Length distribution of unigenes *Tor tambra*.

**Fig. 3:**
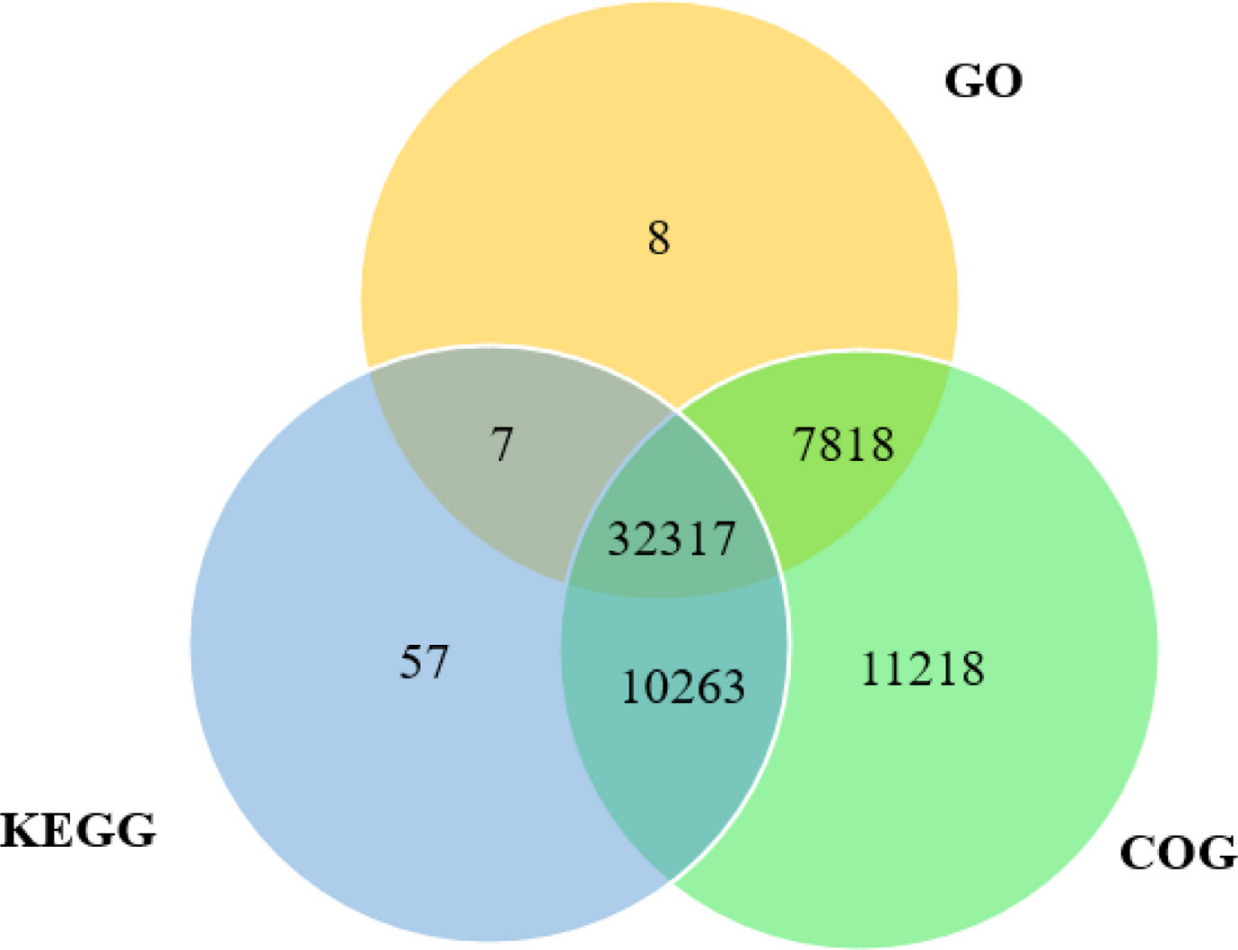
Venn diagram showing differences and commonality of annotation based on GO, KEGG and COG.

**Table 2:** Unigenes functional annotation by various databases.

Database	Number of Unigenes	Percentage (%)
GO	40,150	63.54
KEGG	42,644	67.48
COG	61,616	97.51
Annotated in at least one database	50,405	79.77
Annotated in all database	32,317	51.14
All unigenes	63,191	100.00

**Fig. 4:**
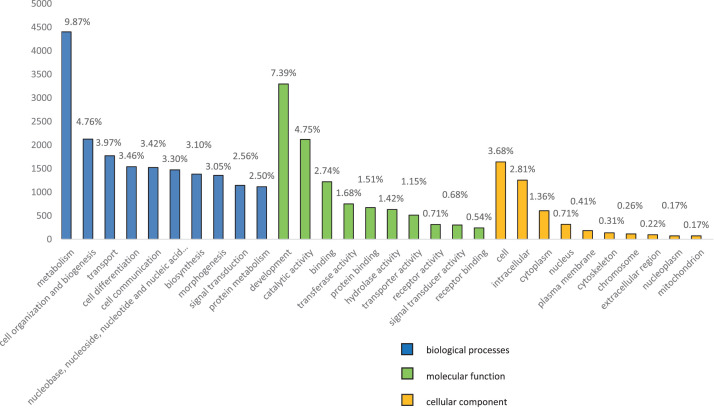
GO functional annotations.

Fig. 4 showed the top ten subcategories account for each main ontology for GO databases. For biological process, 4404 (9.87%) were in the metabolism process, 2125 (4.76%) accounted for cell organization and biogenesis while another 1773 (3.97%) were in transport. For molecular function, 3297 (7.39%) were responsible for development while 2121 (4.75%) and 1222 (2.74%) counts were catalytic activity and binding, respectively. Meanwhile, for cellular component, a total of 1643 (3.68%) counts were accounted for cell, 1256 (2.81%) were categorized as intracellular and cytoplasm with a count of 608 (1.36%). There is a very small number of counts that grouped to extracellular region (0.22%), nucleoplasm (0.17%) and mitochondrion (0.17%).

**Fig. 5:**
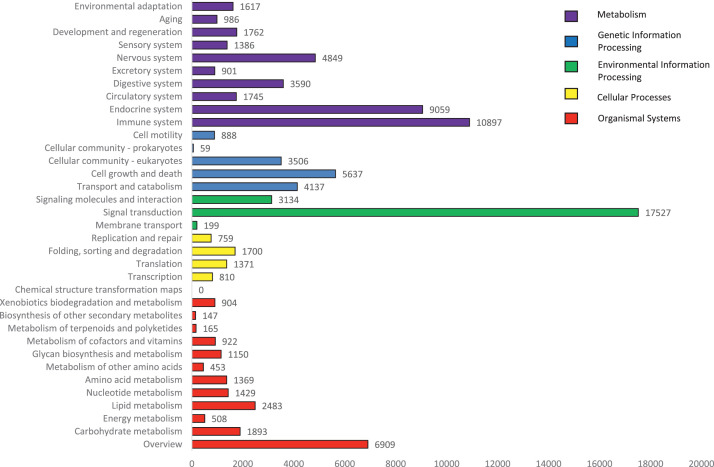
KEGG annotation.

**Fig. 6:**
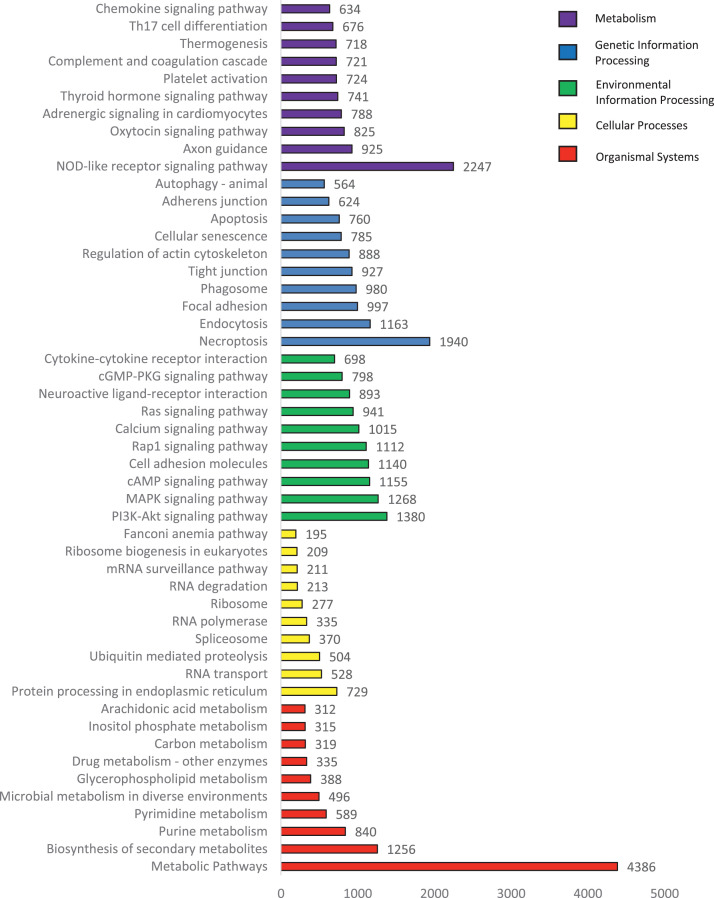
Top 10 KEGG annotations.

KEGG is another widely-used reference database consisting of pathway networks for integrating and interpreting large-scale datasets generated by RNA sequencing. A total of 34 categories of KEGG database consisting of 5 main groups (Cellular Processes, Environmental Information Processing, Genetic Information Processing, Metabolism and Organismal System) had been mapped and successfully located to 304 known KEGG pathways (Fig. 5). Among the five main categories, the largest category was organismal system (36,792, 38.79%) whilst genetic information processing had the lowest count (4640, 4.89%). The cluster having the most counts are as follow: signal transduction (17527, 18.48%), immune system (10897, 11.49%) and endocrine system (9059, 9.55%). In terms of signal transduction, various pathways such as two-component system, MAPK, ErbB, Ras, Rap1, Wnt, Notch, Hedgehog, TGF-beta, Hippo. VEGF, Apelin, JAK-STAT, NF-kappa B, TNF, HIF-1, FoxO, calcium, phosphatidylinositol, phospholipase D, sphingolipid, cAMP, cGMP-PKG, PI3K-Akt, AMPK and mTOR were found in *Tor tambra,* indicating a large number of signal generation during development stage. Fig. 6 shows the top 10 KEGG cluster components with the most counts among the 5 main KEGG groups. The largest count was metabolic pathway from metabolism category (4386, 4.62%), followed by NOD-like receptor signaling pathway (2247, 2.37%) and necroptosis (1940, 2.05%). Necroptosis belongs to the category cellular processes while NOD-like receptor signaling pathway belong to the organismal systems category.

**Fig. 7:**
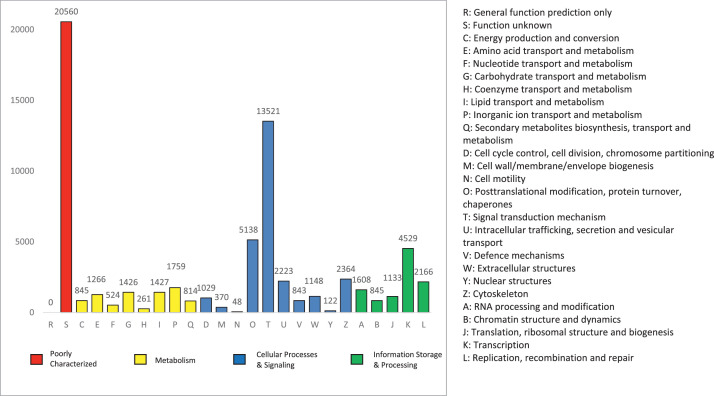
COG annotation.

COG database consists of clusters of orthologous groups and is divided into 25 COG classifications (Fig. 7). Altogether 63,191 unigenes were mapped to COG database that can be grouped into 4 mainly categories, information storage and processing (15.59%), cellular processes and signaling (40.63%), metabolism (12.62%) and poorly characterised (31.17%). Among the 25 classifications, the largest clusters were function unknown (20560, 31.17%) and signal transduction mechanism (13521, 20.50%), followed by posttranslational modification, protein turnover, chaperones (5138, 7.79%), transcription (4529, 6.87%) and cytoskeleton (2364, 3.58%).

73 growth-related genes and 30 immune-related genes were selected based on literature review [4], [5], [6], [7], [8], [9], [10], [11], [12], [13], [14]. Each gene was searched for its respective accession number compatible to its protein sequence in NCBI (https://www.ncbi.nlm.nih.gov/). Out of the 103 genes, 51 growth-related genes and 13 immune-related genes were selected based on a stringent E-value cutoff of 10^−10^. Table 3 had listed on the growth-related proteins while Table 4 listed for immune-related proteins.

**Table 3:** Growth-related protein. Protein marked with * asterisk sign were proteins selected after *e*-value cutoff while best parameters were inputed for proteins that did not pass the cutoff filter.

Contig ID	Protein	Accession	Blast Hit	Aligned Length	Similarity (%)	Reference
TRINITY_DN2932_c0_g1_i4.p1	*Acetyl-CoA carboxylase alpha (Haplotype 1)	ADT82650.1	15	2342	74.552	[7]
TRINITY_DN2318_c0_g1_i1.p1	*Acetyl-CoA carboxylase alpha (Haplotype 2)	ADX43925.1	15	2390	96.987	[7]
TRINITY_DN2932_c0_g1_i4.p1	*Acetyl-CoA carboxylase 2	XP_018962132.1	20	1837	96.788	[7]
TRINITY_DN1703_c0_g1_i1.p1	*Acetyl-CoA Acetyltransferase 2	AAD34966.1	13	395	91.899	[8]
TRINITY_DN1946_c0_g2_i1.p1	*Alpha actinin 3	XP_031588180.1	189	896	93.973	[6]
TRINITY_DN2816_c0_g1_i1.p1	*ATP synthase	BAA82837.1	10	518	99.421	[5]
TRINITY_DN7716_c0_g1_i1.p1	*G2/mitotic-specific cyclin-B1	NP_571588.1	27	397	94.71	[7]
TRINITY_DN7305_c0_g1_i14.p1	*Cell division cycle protein 20	NP_998245.2	460	496	90.524	[7]
TRINITY_DN9513_c0_g1_i1.p1	*Conserved Edge Expressed	ABK35126.2	2	322	91.304	[11]
TRINITY_DN148_c0_g1_i1.p1	*Creatine kinase M-type	XP_028660838.1	18	381	81.102	[6]
TRINITY_DN4485_c0_g1_i10.p1	*Peroxisomal carnitine O-octanoyltransferase	XP_018953213.1	35	333	89.189	[9]
TRINITY_DN7185_c0_g1_i1.p1	*Cathepsin K precursor	NP_001017778.1	54	335	68.657	[5]
TRINITY_DN2821_c0_g1_i10.p1	*Cathepsin L	AAI08032.1	54	612	95.425	[5]
TRINITY_DN3405_c0_g1_i1.p2	*25-hydroxycholesterol_7-alpha-hydroxylase	XP_699028.2	69	506	81.423	[9]
TRINITY_DN2424_c0_g1_i2.p1	*Delta-6_desaturase	AZL94116.1	12	444	97.973	[13]
TRINITY_DN3834_c0_g3_i1.p1	*Elongation of very long chain fatty acids protein 6	XP_018957993.1	25	266	99.624	[5]
TRINITY_DN787_c0_g1_i1.p1	*Fatty acid synthase	ARO92273.1	35	2517	94.915	[9]
TRINITY_DN14382_c0_g1_i1.p1	*Farnesyl pyrophosphate synthase isoform X1	XP_005472704.1	1	359	81.058	[9]
TRINITY_DN11024_c0_g1_i2.p1	*Forkhead box protein K1 isoform X1	XP_025764132.1	90	687	64.338	[9]
TRINITY_DN741_c0_g1_i8.p1	*Glucose-6-phosphatase	AVP32214.1	8	355	95.211	[5]
TRINITY_DN13312_c0_g2_i4.p1	*Growth hormone receptor	ADZ13484.1	29	602	95.085	[10]
TRINITY_DN46810_c0_g1_i2.p1	*Glutathione synthetase	XP_018970229.1	1	475	90.105	[9]
TRINITY_DN909_c1_g1_i3.p1	*70-kDa heat shock proteins	AAF70445.1	49	643	85.07	[8]
TRINITY_DN1380_c0_g2_i1.p1	*Insulin-like growth factor-binding protein 1	ACV72066.1	45	262	96.947	[7]
TRINITY_DN958_c0_g1_i1.p1	*Insulin-like growth factor-binding protein 2	ACM47497.1	54	274	97.08	[7]
TRINITY_DN7562_c0_g1_i3.p1	*Insulin-like growth factor-binding protein 3	ACM47527.1	67	293	89.761	[7]
TRINITY_DN4955_c0_g1_i1.p1	*Inositol Monophosphatase 1	XP_018975133.1	5	282	97.872	[9]
TRINITY_DN230_c0_g1_i10.p1	*Insulin-induced_gene_1_protein	NP_956163.1	8	251	97.61	[8]
TRINITY_DN5482_c0_g1_i1.p1	*Tyrosine-protein kinase JAK2-like	XP_022620925.1	1540	1117	76.455	[14]
TRINITY_DN844_c0_g1_i1.p1	*Lipopolysaccharide binding protein	NP_001118057.1	70	469	74.2	[8]
TRINITY_DN1369_c0_g1_i2.p1	*Hepatic triacylglycerol lipase	XP_018956861.1	23	498	91.767	[5]
TRINITY_DN2357_c0_g1_i12.p1	*Myocyte Enhancer Factor 2°	BAA33567.1	22	475	92.632	[11]
TRINITY_DN31591_c0_g1_i2.p1	*Myostatin 1	AJF48833.1	44	375	98.133	[11]
TRINITY_DN63384_c0_g1_i1.p1	*Myostatin 2	AJF48834.1	43	366	94.536	[11]
TRINITY_DN610_c0_g1_i3.p1	*Cytochrome P450	AAK37960.1	178	505	68.119	[8]
TRINITY_DN26556_c0_g1_i1.p1	*Paired box protein 7 isoform X1	XP_013988550.1	230	520	93.077	[11]
TRINITY_DN6261_c0_g1_i12.p1	*Cytosolic phospholipase A2 gamma-like	XP_018952444.1	45	580	61.724	[5]
TRINITY_DN98343_c0_g1_i1.p1	*Proopiomelanocortin	AAM93491.2	3	117	40.171	[14]
TRINITY_DN10545_c1_g2_i1.p1	*Peroxisome proliferator-activated receptor alpha	CAJ76702.1	175	462	87.013	[8]
TRINITY_DN6218_c0_g1_i1.p1	*Peroxisome proliferator-activated receptor beta	ACR15760.1	1171	488	74.795	[8]
TRINITY_DN2759_c3_g1_i3.p1	*Prolactin receptor	QIB98245.1	45	611	86.579	[10]
TRINITY_DN371_c1_g1_i2.p1	*Antithrombin-III	XP_018920986.1	83	450	91.778	[5]
TRINITY_DN2228_c1_g2_i1.p1	*SMAD family member 3	ABI94729.1	25	423	99.527	[9]
TRINITY_DN2168_c0_g1_i3.p1	*Secreted Protein Acidic And Cysteine Rich	XP_003447656.1	34	300	83.667	[9]
TRINITY_DN10498_c0_g1_i2.p1	*Squalene monooxygenase	NP_001103509.1	3	557	90.305	[9]
TRINITY_DN26296_c0_g1_i1.p1	*Somatostatin Receptor type 1-like	XP_018943223.1	563	367	98.365	[10]
TRINITY_DN17037_c0_g1_i2.p1	*Somatostatin Receptor type 2-like	XP_018946514.1	404	337	88.427	[10]
TRINITY_DN6684_c0_g1_i4.p1	*Signal transducer and activator of transcription 1b	NP_956385.2	28	722	80.332	[14]
TRINITY_DN150_c0_g1_i2.p1	*Signal transducer and activator of transcription 2	NP_001258730.1	29	852	78.638	[14]
TRINITY_DN677_c0_g1_i4.p1	*Signal transducer and activator of transcription 3	BAH47263.1	28	806	99.007	[14]
TRINITY_DN28414_c0_g1_i5.p1	*Signal transducer and activator of transcription 4	NP_001004510.1	29	679	95.582	[14]
TRINITY_DN12936_c0_g1_i1.p1	*Signal transducer and activator of transcription 5	BAH47264.1	28	787	84.117	[14]
TRINITY_DN8483_c0_g1_i1.p1	*Ubiquitin carboxyl-terminal hydrolase 38	XP_003449754.1	114	1039	70.356	[9]

**Table 4:** Immune-related proteins. Protein marked with * asterisk sign were proteins selected after *e*-value cutoff while best parameters were inputed for proteins that did not pass the cutoff filter.

Contig ID	Protein	Accession	Blast Hit	Assembled Length	Similarity (%)	Reference
TRINITY_DN399_c1_g1_i6.p1	*C-X-C Motif Chemokine Receptor 4	BAA32797.1	454	338	93.491	[4]
TRINITY_DN3511_c0_g1_i2.p1	*Myeloid differentiation primary response protein MyD88	XP_018923074.1	18	282	91.489	[14]
TRINITY_DN37340_c0_g2_i1.p1	*60S ribosomal protein L8	XP_034741439.1	4	257	98.054	[4]
TRINITY_DN29928_c0_g1_i1.p1	*Toll-like receptor 1	NP_001124065.1	264	798	75.439	[10]
TRINITY_DN32082_c1_g1_i2.p1	*Toll-like receptor 13	NP_001133860.1	941	951	45.216	[14]
-	*Toll-like receptor 14	AXL48518.1	451	-	-	[14]
TRINITY_DN33713_c0_g1_i2.p1	*Toll-like receptor 2	NP_997977.1	279	787	79.288	[14]
TRINITY_DN10726_c0_g1_i3.p1	*Toll-like receptor 21	AVX48323.1	829	960	87.604	[10]
TRINITY_DN32082_c1_g1_i2.p1	*Toll-like receptor 22	NP_001117884.1	1	958	47.182	[10]
TRINITY_DN39710_c0_g1_i2.p1	*Toll-like receptor 3	ABL11473.1	581	904	88.496	[12]
TRINITY_DN32146_c0_g1_i5.p1	*Toll-like receptor 4ba	AHH85806.1	300	755	73.51	[12]
TRINITY_DN32146_c0_g1_i5.p1	*Toll-like receptor 4bb	AHH85807.1	379	817	91.31	[12]
TRINITY_DN12666_c0_g1_i5.p1	*Toll-like receptor 5b precursor	NP_001124067.2	716	686	79.446	[14]
TRINITY_DN50330_c0_g1_i1.p1	*Toll-like receptor 7	AIS23537.1	550	1026	40.448	[14]
TRINITY_DN28916_c0_g1_i1.p1	*Toll-like receptor 9	ADE20130.1	1252	69	33.333	[4]

## 2 Experimental Design, Materials and Methods

### 2.1 Sampling and RNA extraction

A euthanized juvenile fish fry was provided by a local fish breeder. The whole specimen was homogenized in Wizol reagent (WizBio), a Trizol-like reagent. Total RNA extraction was subsequently performed as per the manufacturer's instructions.

### 2.2 Library construction and sequencing

Approximately 1 ug of total RNA was used as the input for mRNA enrichment using NEBNext Poly(A) mRNA magnetic isolation module (NEB). The enriched mRNA was subsequently processed using the NEBNext Ultra II non-directional RNA library preparation kit (NEB). Sequencing of the RNA library was performed on an Illumina NovaSeq6000 using the run configuration of 2 × 150 bp.

### 2.3 Sequence data processing and assembly

Raw reads were filtered for poly-G at the 3’ end, Illumina adapter and low-quality reads using the default setting of fastp v0.22.0 [15]. The trimmed paired-end reads were assembled *de novo* using Trinity v.2.8.5 using the default setting [16]. The transcriptome completeness was assessed using BUSCO v5 [17] based on the single-copy orthologs represented in the actinopterygii_odb10 database.

### 2.4 Mitogenome reconstruction and phylogeny

Trimmed pair-end reads were aligned to the reference mitochondrial genome of the Javan mahseer (GenBank Accession Code: NC_036511.1) using bowtie2 [18]. The SAM alignment was normalized to reduce high coverage particularly in the rRNA gene region followed by consensus generation using the samtools mpile up and bcftools [19]. The draft mitogenome assembly was annotated and used for phylogenetic analysis as previously described [1].

### 2.5 Annotation of unigenes

The protein coding sequences were extracted using TransDecoder v.5.5.0 followed by clustering at 98% protein similarity using cdhit v4.7 (-g 1 -c 98). The non-redundant predicted protein dataset was annotated using eggNOG mapper (evolutionary genealogy of genes: Non-supervised Orthologous Groups) with a minimum *E*-value of 0.001. Functional annotation of unigenes was executed by mapping against the three databases, GO (Gene Ontology), KEGG (Kyoto Encyclopedia of Genes and Genomes) and COG (the Clusters of Orthologous Groups).

## Ethics Statement

All experiments comply with the ARRIVE guidelines and were carried out in accordance with the U.K. Animals (Scientific Procedures) Act, 1986 and associated guidelines, EU Directive 2010/63/EU for animal experiments, or the National Institutes of Health guide for the care and use of Laboratory animals (NIH Publications No. 8023, revised 1978).

## CRediT authorship contribution statement

**Melinda Mei Lin Lau:** Writing – original draft, Data curation, Conceptualization. **Leonard Whye Kit Lim:** Data curation, Writing – original draft, Conceptualization. **Hung Hui Chung:** Conceptualization, Funding acquisition, Writing – review & editing. **Han Ming Gan:** Methodology, Conceptualization, Writing – review & editing.

## Declaration of Competing Interest

The authors declare that they have no known competing financial interests or personal relationships which have or could be perceived to have influenced the work reported in this article.
